# A Manual of the Diseases of the Eye, or a Treatise on Opthalmology

**Published:** 1846-08

**Authors:** 


### PART III.—BIBLIOGRAPHICAL NOTICES.

ARTICLE VI.

*A Manual of the Diseases of the Eye, or a Treatise on Opthalmology.* By S. Littell, Jr., M. D., one of the Surgeons of the Wills Hospital, Fellow of the College of Physicians of Philadelphia, etc. etc. Second edition revised and enlarged. Philadelphia: Hogan & Thompson. 1846. pp. 372. 12 mo.

Dr. Littell has, in the clearest manner, condensed in this small volume, all that is of practical value in relation to the diseases of the eye, without forfeiting its name to the title of a Manual. We can with much satisfaction commend this treatise to the favorable notice of the profession. The physician will find it eminently valuable as a book of reference, and, from the clearness of its style, it will be equally valuable to the student.

The Institution to which Dr. Littell is attached as surgeon is exclusively for the blind and lame, and this, as well as an extensive private practice in diseases of the eye, renders him entirely qualified to write on this subject.

The first edition was noticed with commendation by a British Journal of high standing, and the work was republished in London. There is a copious glossary compiled by the author and his English editor. H. S. H.

